# Supplementary material for: Gray whale transcriptome reveals longevity adaptations associated with DNA repair and ubiquitination
Source: Aging Cell. 2020 Jun 9;19(7):e13158. doi: 10.1111/acel.13158 (PMC7433004; doi:10.1111/acel.13158)
Supplement: Supplementary file 1 — Supplementary Material [file ACEL-19-e13158-s001.docx]

**Gray whale transcriptome reveals longevity adaptations associated with DNA repair, autophagy and ubiquitination.**

Vadim Fraifeld

**Supplementary Information:**

**This PDF file includes:**

Fig. S1. Heatmap representation of correlation coefficients between GO expression ranks of mammalian species.

Table S1. The 20 top-expressed unannotated genes (contigs with a high-count number and a sequence length comparable to the size of common mRNAs) in the *de novo* transcriptome of the gray whale.

**Other supplementary materials for this manuscript include the following:**

Datasets S1 to S3:

Dataset S1 – Full list of unknown genes from the gray whale transcriptome.

Dataset S2 – List of contigs with TPM in the gray whale transcriptome.

Dataset S3 – TopGO enrichment results.

Dataset S4 – Rank values for all species examined.

Dataset S5 – List of LAGs found in the gray whale transcriptome.


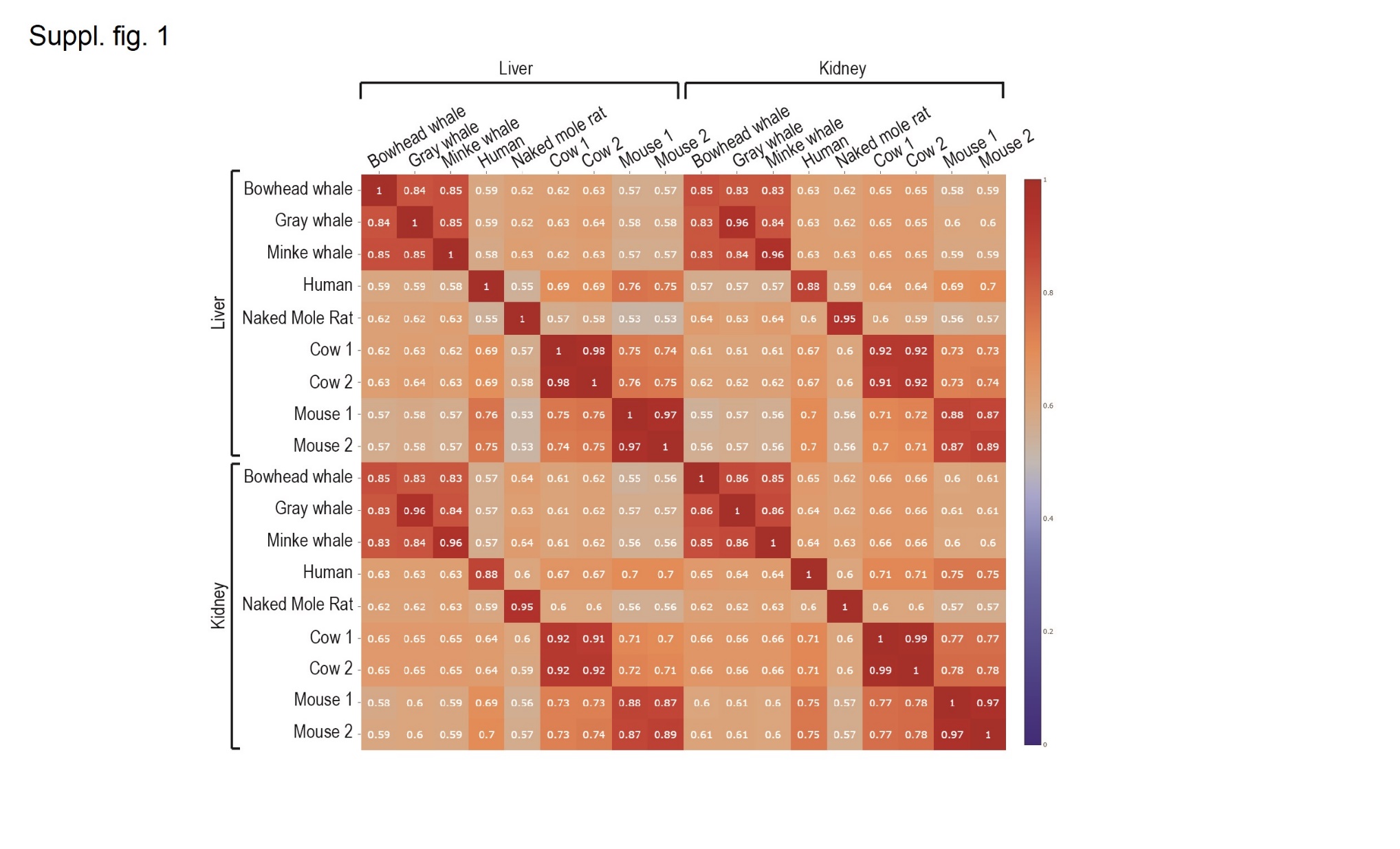


Fig. S1. Heatmap representation of Spearman’s correlation coefficients between GO expression ranks of mammalian species. In the figure, correlations are shown as a matrix heatmap to compare values across seven mammalian species and two tissues. The red to blue gradient scale indicates differences between high and low correlation coefficients. The protein-coding transcripts, from all transcriptomes, are grouped by GOs and their composite TPMs are computed within each GO term. Terms are ranked based on the composite expression from 1 to 9,779 (number of GO terms in the gray whale’s transcriptome). The correlation shown in each cell of the matrix represents the Spearman score, between all ranked GO terms for two species.

Table S1. The 20 top-expressed unannotated genes (contigs with a high-count number and a sequence length comparable to the size of common mRNAs) in the *de novo* transcriptome of the gray whale. Contig counts were normalized as TPMs (transcripts per million). Length of genes is given in base pairs (bp). BLAST search was performed for each sequence, followed by manual investigation of the resulted sequences (domain/motif search, checking existing literature for each sequence, etc.) and the most informative results were included in the table.

| **Contig ID** | **Liver TPM** | **Kidney TPM** | **Length bp** | **Manual curation** | **Selected Categories** |
| --- | --- | --- | --- | --- | --- |
| c19144_g2_i1 | 5073 | 7750 | 458 | Predicted, hypothetical, Zinc fingers | Binding, regulation, metabolic process |
| c31983_g1_i1 | 1920 | 914 | 865 | Ribosomal RNA | Mitochondria, ATP |
| c71268_g1_i1 | 1540 | 121 | 692 | Predicted, phenylalanine hydroxylase (PAH) | Biogenic amine synthesis, oxidation-reduction process |
| c8949_g1_i1 | 1500 | 173 | 630 | Predicted, serpin family F member 2 (SERPINF2) | Formation of fibrin clot (clotting cascade) |
| c71306_g1_i1 | 984 | 1111 | 400 | Predicted, actin, beta (ACTB) | ERK signaling and cytoskeleton remodeling, ATP binding, protein kinase binding |
| c7678_g1_i1 | 958 | 117 | 777 | Predicted, CYP1A1 mRNA for cytochrome 1A1 | Iron/heme binding, oxidation-reduction process, lipid hydroxylation, amine metabolic process |
| c19059_g3_i1 | 915 | 499 | 345 | Predicted, GCLC, DOB, MDR/TAP, TAP2, PSMB8 genes, TAP1 | Ferroptosis and Metabolism, protein heterodimerization activity and coenzyme binding |
| c19059_g3_i8 | 795 | 403 | 385 | Predicted, histocompatibility complex class II | immune response, positive regulation of T cell mediated cytotoxicity, peptide antigen binding |
| c14657_g2_i1 | 773 | 256 | 412 | Predicted, calreticulin (CALR) | Ubiquitin protein ligase binding, endoplasmic reticulum, nuclear envelope, protein binding and folding |
| c16102_g1_i1 | 547 | 421 | 1143 | Predicted, uncharacterized | N/A |
| c16992_g2_i1 | 472 | 225 | 1137 | Ribosomal RNA | Ribosome |
| c15316_g1_i1 | 407 | 38 | 398 | Partial APOA2 gene for apoliprotein A2 | Cholesterol homeostasis, lipoprotein metabolic process, chylomicron |
| c18931_g5_i1 | 308 | 634 | 448 | Predicted, peroxiredoxin 3 (PRDX3) | Detoxification of reactive oxygen species, mitochondrion, cellular oxidant detoxification, cellular response to oxidative stress |
| c32840_g1_i1 | 279 | 89 | 389 | Predicted, G protein pathway suppressor 2 (GPS2) | Regulation of transcription by RNA polymerase II, nucleus, B cell differentiation |
| c58631_g1_i1 | 268 | 952 | 737 | Predicted, N-myc downstream regulated 1 (NDRG1) | Apoptosis and autophagy, cytoplasm, signal transduction, mast cell activation, peripheral nervous system myelin maintenance |
| c11859_g1_i2 | 246 | 166 | 452 | Predicted, CAAX box protein 1-like, retrotransposon Gag-like protein 8 | Proliferation, differentiation, carcinogenesis and apoptosis |
| c47222_g1_i1 | 218 | 144 | 597 | Predicted, cellular repressor of E1A-stimulated genes 1 (CREG1) | Innate Immune system and senescence and autophagy in cancer, promote cellular proliferation and inhibit differentiation |
| c32069_g1_i1 | 207 | 1120 | 1289 | Predicted, GABA type A receptor associated protein like 1 (GABARAPL1) | Autophagy pathway, microtubule binding and GABA receptor binding |
| c2003_g1_i1 | 165 | 60 | 1489 | Predicted, translation initiation factor IF-2-like, isolate yakQH1 | N/A |
| c13320_g1_i1 | 194 | 423 | 1223 | Predicted, prothymosin alpha (PTMA), mRNA | Immune response |
